# Speech comprehension in noise—considerations for ecologically valid assessment of communication skills ability with cochlear implants

**DOI:** 10.1007/s00106-022-01232-3

**Published:** 2022-12-08

**Authors:** Matthias Hey, Alexander Mewes, Thomas Hocke

**Affiliations:** 1 Department of Otorhinolaryngology, Head and Neck Surgery, Audiology, UKSH, Campus Kiel, Arnold-Heller-Straße 14, 24105 Kiel, Germany; 2 Cochlear, Hannover, Germany

**Keywords:** Hearing aids, Speech reception threshold, Speech audiometry, Cochlear implant, Reliability and validity

## Abstract

**Background:**

Nowadays, cochlear implant (CI) patients mostly show good to very good speech comprehension in quiet, but there are known problems with communication in everyday noisy situations. There is thus a need for ecologically valid measurements of speech comprehension in real-life listening situations for hearing-impaired patients. The additional methodological effort must be balanced with clinical human and spatial resources. This study investigates possible simplifications of a complex measurement setup.

**Methods:**

The study included 20 adults from long-term follow-up after CI fitting with postlingual onset of hearing impairment. The complexity of the investigated listening situations was influenced by changing the spatiality of the noise sources and the temporal characteristics of the noise. To compare different measurement setups, speech reception thresholds (SRT) were measured unilaterally with different CI processors and settings. Ten normal-hearing subjects served as reference.

**Results:**

In a complex listening situation with four loudspeakers, differences in SRT from CI subjects to the control group of up to 8 dB were found. For CI subjects, this SRT correlated with the situation with frontal speech signal and fluctuating interference signal from the side with R^2^ = 0.69. For conditions with stationary interfering signals, R^2^ values <0.2 were found.

**Conclusion:**

There is no universal solution for all audiometric questions with respect to the spatiality and temporal characteristics of noise sources. In the investigated context, simplification of the complex spatial audiometric setting while using fluctuating competing signals was possible.

## Speech comprehension in realistic hearing situations

In the clinical care of hearing-impaired patients with hearing systems, there is a need for measurements of speech comprehension—especially in follow-up—that cover various aspects of everyday use. By means of audiometric examinations in the clinic or practice, a realistic model of typical daily communication situations is created. The results obtained in this way can be used for the adequate assessment of the hearing deficiencies resulting from various hearing pathologies. Important characteristics of everyday listening situations include fluent speech presentation, multimodal stimuli, realistic speech levels, spatial separation of signal and noise sources, reverberation and the occurrence of direct and reflected sound, i.e., competing noise, and the acoustic characteristics of different speakers [8, 25, 41].

Patients with a severe degree of hearing loss who can no longer achieve sufficient speech comprehension with a hearing aid have the option of (re)gaining hearing through a cochlear implant (CI). After this surgical therapy, speech understanding must be checked regularly. This serves to characterize the hearing handicap relevant to everyday life in order to optimize understanding in the future. It is generally accepted that speech understanding in quiet and in noise provides suitable surrogate parameters for describing the ability to communicate in everyday life [9, 28].

The inventory of methods available for this purpose consists of established examination methods such as the Freiburg Monosyllabic Word Test in quiet [14, 22, 23]. The assessment of results in the context of other functional diagnostic procedures is supported by more than half a century of clinical experience [14, 29]. Typical applications of the Freiburg Monosyllabic Word Test are the diagnosis of hearing disorders, the diagnosis of hearing loss [32], the determination of an indication for hearing aids [9], and the subsequent assessment of their success [16, 22, 23] and use as a target parameter in pharmacological studies [35]. As has been shown in the context of CI provision, monosyllabic word tests can be used to identify factors influencing therapy [4, 19] and for the individual prognosis of postoperative speech understanding [13, 22]. These tests are usually carried out under standardized test conditions, i.e., measurement in free sound field with frontal sound or headphone presentation [3, 7].

Certain supplementary investigations appear to be especially suitable for answering particular questions regarding speech comprehension in competing noise [39, 41]. Here, the audiological methods were extended by alternative spatial loudspeaker arrangements [7, 30, 39, 44] and various kinds of interfering signals [11, 20]. In initially exclusively scientific investigations, an experimental setup with increased methodological effort is justifiable. In the context of patient care, the space as well as personnel resources in functional diagnostics may limit the number and scope of tests to be performed. Coping with a standardized measurement protocol can also be a burden for some patients [23].

Further developments in CI processor technology have improved speech intelligibility in noisy environments [10, 17, 30]. However, clinical implementation shows that CI users can benefit from a more individual adjustment of their CI systems beyond the established level [18, 23, 34, 38]. This results in new test conditions, which, however, are associated with increased time expenditure for the patient and the clinic. The fitting method proposed by Rader et al. [38], for example, offers a useful approach to counteracting the increased workload for the clinic, enabling the workload of the clinic staff to be reduced by the active and independent cooperation of the patient [34]. The same applies to the simple speech audiometric test procedures based on mobile devices, as described recently [26]. For more complex hearing situations, this workload reduction is currently only possible to a limited extent. For example, in the investigation of CI noise suppression algorithms with directional hearing, the assessment of hearing improvement via the required setup with more than two loudspeakers [10, 17, 30] is not possible everywhere. The microphone characteristic ForwardFocus [17] is an algorithm developed for the so-called cocktail party situation [36]. Improved speech intelligibility was described here especially for demanding (realistic) listening situations [21]. For these listening situations, an audiometric setting was created for measuring speech understanding in fluctuating background noise, which has a high ecological validity [25, 41]. This is characterized by the use of fluent speech my means of a sentence test. Furthermore, spatially distributed signal sources with frontal speech presentation and competing noise from several non-coherent sources in the posterior hemisphere contribute to a realistic setting. The fluctuating interfering signal is ICRA (International Collegium of Rehabilitative Audiology) noise [11], which simulates male speakers with sound coming from several directions. The results of the CI patients are compared with those of normal-hearing people [39]. This was done by using a reference metric introduced elsewhere [15, 17].

In the present study we investigated whether, and to what extent, such complex listening situations may be simplified to measure speech comprehension in noise. This simplification is to be considered in two dimensions. For this purpose, different temporal characteristics of the noise were chosen, with stationary speech-simulating Oldenburg noise compared with fluctuating ICRA noise. Also, simplified spatial arrangements of the noise source(s) were investigated, using load speaker constellations with two or only one signal source compared with the reference configuration with four speakers. The results for speech reception threshold (SRTs), measured with the Oldenburg Sentence Test (OLSA), are compared in different audiometric settings and evaluated with respect to their equivalence. In addition, the use of two generations of speech processors was investigated, to determine the extent to which the technical development of these can contribute to improved speech understanding in these complex listening situations.

## Methods

### Patients

A group of 20 CI patients with postlingual deafness participated in this study. The study was approved by the local ethics committee (D 06/18). All examinations were conducted in accordance with the ethical standards of the Institutional and National Research Commission and the 1964 Declaration of Helsinki and its subsequent amendments, or with comparable ethical standards.

The inclusion criteria for the adult participants in the study were postlingual onset of hearing loss and use of a CI24RE or CI5xx cochlear implant (Cochlear Limited, Australia) with full insertion of the electrode array into the scale tympani. Participants were to achieve a speech understanding of at least 80% at the initial examination using the OLSA in quiet (65 dB_SPL_). Bilateral implantation was not an exclusion criterion, but only one ear per patient was examined in this study. In 17 patients all 22 electrodes were activated, in three cases 21 electrodes were active.

The mean age of the participants was 53 years (minimum: 31 years, maximum: 76 years). Participants had a mean CI experience of 8.3 years (minimum: 6.0 years, maximum: 15.4 years). The biographical details of the participants have been published elsewhere [15].

To relate the CI patients’ speech comprehension to comparable data for normal-hearing adults, ten normal-hearing adults were additionally recruited and examined monaurally in all test conditions. The opposite ear was passively masked by means of earplugs and capsule headphones. A tone-audiometric examination was performed for each of these participants to ensure that they had normal hearing according to DIN ISO 8253:3 [24] in the frequency range of 250–8000 Hz. For comparisons between impaired and normal hearers, the mean speech-comprehension value for the normal hearers was subtracted in each case from the respective value of the hearing-impaired person.

### Test procedure

In this study, repeated measurements were performed on the same individuals. The tests were performed in three test sessions (randomized order) 2–3 weeks apart to allow subjects to accustom themselves to different speech processors and signal-processing algorithms under everyday conditions. For testing, the OLSA was used throughout.

All tests were performed in an acoustically shielded audiometric booth (ISO 8253:227). The loudspeakers were located 1.3 m from the patient. The following speaker configurations were used:

- S0N0—Speech and noise from front
- S0N90—Speech frontal and noise 90° ipsilateral to the examined CI
- S0N90, 180, 270—Speech frontal and interfering sound from 90°, 180° and 270° simultaneously

Sentences in noise were presented by using a computer-based implementation of the OLSA (Equinox audiometer, Interacoustics, Denmark, and evidENT 3 software, Merz Medizintechnik, Germany). The Oldenburg sentences [43] were presented at a constant noise level of 65 dB_SPL_. The noises used were the stationary speech-simulating Oldenburg noise on the one hand and the fluctuating ICRA noise on the other [11]. For the latter, track no. 5 of the ICRA CD was used, which has the spectral and temporal characteristics of a single male speaker. For noise presentation from the posterior hemisphere (S0N90, 180, 270), the noise from the different directions was presented non-coherently. The SRT was measured by using an adaptive method [5] and was defined as the signal-to-noise ratio (SNR) that resulted in 50% correct word understanding. All CI users were accustomed to the adaptive testing procedure, having been tested five or more times in our routine clinical practice beforehand. To ensure sufficient reduction of the procedural learning effect, additional training was given at the beginning of each test session (30 sentences at 65 dB_SPL_). The measurement of speech understanding was always monaural. The contralateral CI was switched off for the measurement procedure or contralateral residual hearing was passively deafened by using earplugs and capsule headphones.

At each examination appointment in the clinic, the CI speech processors were checked technically and if necessary, system components were replaced.

All patients used the ACE coding strategy with individually adjusted stimulation rate and number of maxima. The individual map parameters (T and C level) of the CI speech processors were used unchanged throughout the study period. However, the algorithms of the acoustic signal preprocessing were changed according to the study protocol. In each case, the signal processing was used that was activated by the scene classifier in noise [30]: CP910 with the microphone characteristic Beam (CP9Beam) and CP1000, also with Beam (CP10Beam; [40]). These were compared with the manual setting in the CP1000 processor using ForwardFocus (CP10FF). In addition, the signal preprocessing ADRO (automatic dynamic range optimization), ASC (automatic sensitivity control), and SNR-NR (noise reduction) were always activated [30, 33]. After a 2–3-week adaptation period with the respective speech processor, the audiometric tests were performed.

### Data evaluation

To visualize the hearing deficit relevant to everyday life, the SRTs of CI users were plotted relative those of normal-hearing people in the same situation [15, 17]. To compare the different measurement conditions, pairwise intra-individual comparative analyses with Bonferroni correction were performed. A significance level of 0.05 was used to determine significance for two-sided analyses. The results are presented as boxplots.

## Results

**Fig. 1:**
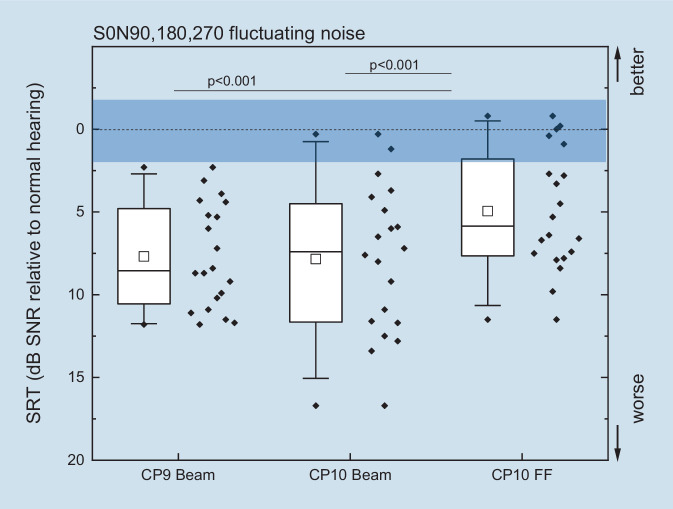
Speech reception thresholds (*SRTs*) measured with the Oldenburg Sentence Test in fluctuating noise (ICRA noise, International Collegium of Rehabilitative Audiology) with frontal presentation of speech and with noise from three loudspeakers in the posterior hemisphere (90, 180, and 270°) and for different cochlear implant (CI) processor configurations. The SRTs of CI patients are presented relative to the speech understanding of normal-hearing persons under the same acoustic conditions (see text, “Patients”; the *shaded* area shows the range for normal hearers). Boxplots with medians (*solid center line*), 25th and 75th percentiles (*box limits*), and the 5th and 95% percentiles (*whiskers*) are shown. The mean is shown as a *square*. Outliers (those falling outside the 5%/95% percentiles) are shown as *diamonds*. Individual results are shown to the *right of each box*. *CP9* speech processor CP9, *CP10* speech processor CP10, *FF* microphone characteristic ForwardFocus, *SNR* signal-to-noise ratio

All study participants were able to complete the tests in noise successfully. The speech understanding measured in the most complex listening situation of this study is shown in Fig. 1. The SRT in fluctuating noise is plotted for the loudspeaker setup S0N90, 180, 270 as a function of the speech processor and its setting relative to the monaural speech understanding of normal-hearing people. A significant improvement of ≈3 dB SNR was found for the CP10 speech processor when switching from beam to ForwardFocus with frontal speech presentation and fluctuating noise from the rear hemisphere. When using the CP10 speech processor, some CI patients were able to reach the monaural reference range of normal-hearing listeners. For comparison with the results for CI users, the mean values and standard deviations of the SRT of the reference collective are listed in Table 1.

**Table 1:** Mean values and standard deviations of the SRT of the Oldenburg Sentence Test in noise for a reference group of 10 normal-hearing persons for different loudspeaker configurations and stationary or fluctuating noise. The investigation was carried out monaurally in free sound field

Measurement condition	Mean value of the SRT (dB SNR)	SD of SRT (dB SNR)
S0N0 stationary noise	−8.2	1.8
S0N0 fluctuating noise	−26.0	2.6
S0N90 stationary noise	−8.1	2.0
S0N90 fluctuating noise	−24.9	3.5
S0N90, 180, 270 stationary noise	−2.2	0.9
S0N90, 180, 270 fluctuating noise	−18.1	1.9

*SD *standard deviation, *SRT* speech reception threshold, *SNR* signal-to-noise ratio

**Fig. 2:**
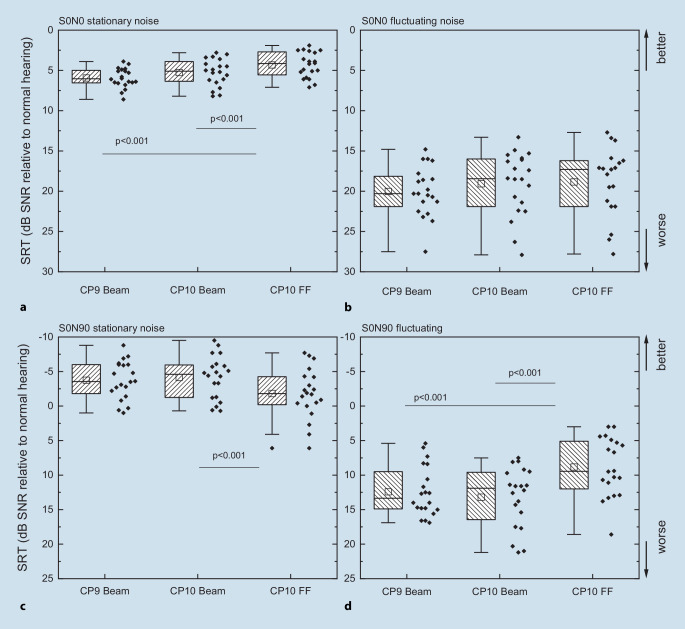
Speech reception thresholds (*SRTs*) measured with the Oldenburg Sentence Test in stationary noise (**a**,**c**) and in fluctuating noise (**b**,**d**) for the loudspeaker setup S0N0 (**a**,**b**) and S0N90 (**c**,**d**) for different cochlear implant processor configurations. *CP9* speech processor CP9, *CP10* speech processor CP10, *FF* microphone characteristic ForwardFocus, *SNR* signal-to-noise ratio

The three speech processor configurations investigated were further examined with reduced spatial loudspeaker setups (S0N0 and S0N90) in stationary and fluctuating noise (Fig. 2), whereby the speech was always presented from the front. The results showed better speech understanding in the stationary noise compared with the fluctuating noise. The majority of CI patients were able to achieve an even better understanding in S0N90 in stationary noise than that attained by normal-hearing persons.

**Fig. 3:**
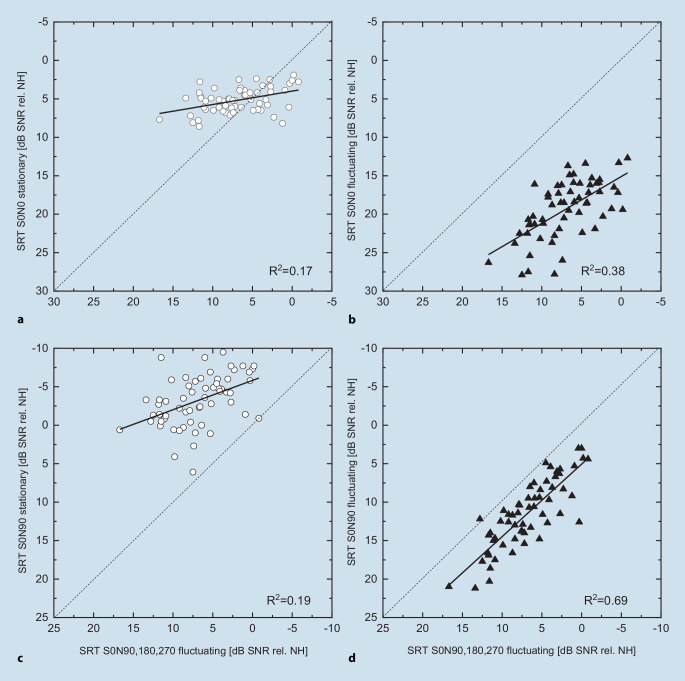
Scatterplot of speech understanding in stationary noise (**a**,**c**) and in fluctuating noise (**b**,**d**), as well as in the speaker setups S0N0 (**a**,**b**) and S0N90 (**c**,**d**) as a function of speech understanding in fluctuating noise (S0N90, 180, 270). The *diagonals* are shown *dotted. NH* normal hearing,* SNR* signal-to-noise ratio, *SRT* speech reception threshold

In Fig. 3, the SRT in the ecologically valid situation S0N90, 180, 270 in fluctuating noise is plotted as a function of comprehension in the other study setups (see Fig. 2). The correlation with speech understanding in stationary noise was low, with *R*^2^ = 0.17 (S0N0) and 0.19 (S0N90). The correlation with understanding in fluctuating noise was much stronger, with *R*^2^ = 0.38 (S0N0) and showed the highest value for the loudspeaker configuration S0N90 of *R*^2^ = 0.69. In this case, the regression line was largely parallel to the angle bisector.

## Discussion

### Methodology of speech audiometry

The starting point of this study was a complex audiometric setup to investigate speech comprehension in fluctuating and spatially separated noise (S0N90, 180, 270). The use of more than one loudspeaker to capture benefit through signal preprocessing in CI systems is an established method [6, 45]. In addition, the ecological validity for a specific everyday situation can be increased by selecting an appropriate interfering noise. In this way, the characteristics of noisy everyday listening situations, such as a family celebration or a visit to a restaurant, can be reproduced [25, 36, 41]. Here, we investigated whether and to what extent this measurement setup may be simplified. A four-speaker setting with fluctuating interfering signals from several noncoherent sources in the rear hemisphere served as a basis. This test setup is not part of the standard measurements of clinical speech audiometry. It can be simplified in two ways: by changing the spatial location and number of loudspeakers and by selecting an appropriate interfering signal. The complex spatial setting was simplified by using two loudspeakers (S0N90) for frontal sound presentation of speech and noise (S0N0) utilizing one loudspeaker only. In addition, the fluctuating ICRA 5 noise signal, which has the spectral and temporal characteristics of a single speaker, was compared with the clinically established stationary speech-shaped noise signal (noise of the OLSA).

Figure 3a and c show that the interfering signal representative of a cocktail party situation cannot be replaced by a stationary noise. The SRTs show only a weak correlation with the reference setting (S0N90, 180, 270) for the signal source configuration S0N0 as well as for S0N90. However, if the competitive signal is retained and only the loudspeaker configuration is simplified to S0N90, a high degree of correlation with the reference setting is shown. A reduction of the methodological effort to determine speech understanding in an ecologically valid listening situation is therefore possible within certain limits. The correlation of the results in Fig. 3 in the individual settings shows that the loudspeaker arrangement can be reduced from four to two. On the other hand, a change from fluctuating to stationary noise is not advisable.

The use of fluctuating interfering signals is suitable to represent everyday listening situations in audiometry [39, 44]. The “currently used speech audiometric methods take into account largely standardised conditions” [31], but the comparatively low degree of complexity limits their ability to represent everyday situations (“ecological validity”; [31]). Despite the relevance of complex situations for everyday communication, which was described very early [36] for hearing in everyday life, the use of more complex interfering signals is not widespread in clinical routine. It can be assumed that the use of fluctuating interfering signals counteracts a standardization in speech audiometry; this trend is certainly desirable, since no signal has currently proven to be universally applicable. So far, depending on the scientific question, a whole spectrum of different signals have been used [11, 39, 44]. Only the use of complex competitive signals enables the assessment of target values for describing or improving speech comprehension in demanding listening situations [37, 44]. It is therefore encouraging to see proposals from various research groups that could promote standardization [11, 12].

### Reference to normal hearing

Dealing with complex listening situations is characterized by special and technically demanding methodology. In addition, results obtained by any particular research group are often difficult for another group to interpret. This can be improved by using normal-hearing individuals as a reference, as was done in this and other studies [15, 17, 39, 44].

The SRT of CI users in relation to normal-hearing subjects is shown in Fig. 2. For the CI users, very low SRTs were found in the setting S0N90 with stationary noise, compared with the control group with normal hearing. This is an artificially created test setting of an everyday hearing situation. The aim here was to map the improvement for the patients through suitable signal preprocessing by means of audiometric tests. The use of beamformers here led a very impressive improvement in SRT in this special situation, even to the extent that the majority of CI users showed better understanding than normal-hearing people. The reason for this is the ideal suitability of the beamformer for this audiometric setup. This argument does not question the benefit of the beamformers, but in the context of the known problems of CI patients in noisy environments [1, 42] this result raises doubt concerning the ecological validity of this particular measurement setup, i.e., S0N90 with stationary noise. This contradiction was recently pointed out by Badajoz-Davila and Buchholz [2]: “...standard speech-in-noise tests overestimate the performance of cochlear implant recipients in the real world. To address this limitation, future assessments need to improve the realism over current tests by considering the realism of both the speech and the noise materials.” In this respect, concerns about the use of stationary noise are justified, especially in discussions about the use of relatively complex noise signals to describe ecologically valid hearing situations [31]. However, the discussion regarding the highest possible ecological validity should not be limited to the complexity of a (test) situation or the signals used in it. It is also, and above all, related to the listening environment of the different people using a CI. For one patient, this may be determined by the noise of a stationary motor, while others are more likely to consider a quiet environment as their daily reality. In their paper, Oberhoffner et al. also point out, among other things, that listening habits and environments change with age. Furthermore, in our opinion, it has not yet been conclusively clarified to what extent the meaningless sentences of the OLSA represent a realistic depiction of the reality of life of our patients. This issue in particular should be the subject of further research.

### Aims of audiometry

Within the framework of audiometric diagnostics, a distinction can be made between the following:

1. Audiometric procedures to diagnose a hearing/understanding deficit and to describe the extent and localization of damage. These do not necessarily have to be ecologically valid. They are intended to support a therapeutic decision.
2. Follow-up monitoring during therapy [9, 27] with the aim of monitoring the development over time as rehabilitation progresses. This aims not only to document development over time, but also to achieve early detection of possible pathologies to achieve therapy goals.
3. Audiometric procedures to address further scientific/clinical questions. These can serve to optimize the communication ability of the affected patients in their everyday situations. They should be oriented closely toward the acoustic everyday reality of these patients and thus have the highest possible ecological validity. It is precisely everyday reality that implies a constant change of these methods. Both the everyday life of the patients and the expanded technical and medical options determine the methodology.

The current discussions on ecological validity go back to the beginning of German-language audiometry: “In recent years and decades, the development of physics and technology has put a wealth of new diagnostic and therapeutic possibilities in the hands of the physician. [...] In the field of acoustics, audiometry has today developed into a fine, indeed the very finest, diagnostic instrument. Even the correct handling of pure-tone audiometry requires knowledge and a lot of practical experience. The situation is somewhat more complicated with speech audiometry, which on the one hand has the advantage that it can be used to measure the entirety of the hearing of complex sounds, but on the other hand has the difficulties of a measuring method the results of which are influenced by a large number of factors. Despite the greater expense of equipment and expertise, this method is indispensable today both for the assessment of hearing ability in general and of changes in hearing caused by therapeutic interventions—in particular, for the fitting of hearing aids” (translated from [46]). This quotation from Zöllner, which is over 60 years old, has lost none of its topicality. It underlines the need for continual improvement, to develop the best possible diagnostic methods and therapy. In the context of ever more highly developed procedures in ENT medicine, methods in speech audiometry must be reconsidered and revised again and again.

## Practical conclusion

- In the diagnosis of hearing disorders that accompanies therapy, there is no universal solution for all audiometric questions.
- In addition to the established standard procedures such as the Freiburg Monosyllabic Word Test and the sentence tests in stationary noise used in German-speaking countries, new tests adapted to the special questions and therapeutic procedures are necessary.
- A complex audiometric setting for speech understanding, consisting of four loudspeakers with a fluctuating masker in the posterior hemisphere, can be reduced to two loudspeakers while retaining the fluctuating masker; this leads to comparable audiometric results in the context studied here.

## Declarations

All procedures performed in studies involving human participants or on human tissue were in accordance with the ethical standards of the institutional and/or national research committee and with the 1975 Helsinki declaration and its later amendments or comparable ethical standards. Informed consent was obtained from all individual participants included in the study.

The supplement containing this article is not sponsored by industry

## References

[1] Abdel-Latif KHA, Meister H (2022). Speech recognition and listening effort in cochlear implant recipients and normal-hearing listeners. Front Neurosci.

[2] Badajoz-Davila J, Buchholz JM (2021). Effect of test realism on speech-in-noise outcomes in bilateral cochlear implant users. Ear Hear.

[3] Batsoulis C, Lesinski-Schiedat A (2017). Sprachaudiometrie in der Begutachtung des Hörvermögens. HNO.

[4] Blamey P, Artieres F, Başkent D, Bergeron F, Beynon A, Burke E, Dillier N, Dowell R, Fraysse B, Gallégo S, Govaerts PJ, Green K, Huber AM, Kleine-Punte A, Maat B, Marx M, Mawman D, Mosnier I, O’Connor AF, O’Leary S, Rousset A, Schauwers K, Skarzynski H, Skarzynski PH, Sterkers O, Terranti A, Truy E, Van De Heyning P, Venail F, Vincent C, Lazard DS (2012). Factors affecting auditory performance of postlinguistically deaf adults using cochlear implants: an update with 2251 patients. Audiol Neurotol.

[5] Brand T, Kollmeier B (2002). Efficient adaptive procedures for threshold and concurrent slope estimates for psychophysics and speech intelligibility tests. J Acoust Soc Am.

[6] Büchner A, Schwebs M, Lenarz T (2020). Speech understanding and listening effort in cochlear implant users—microphone beamformers lead to significant improvements in noisy environments. Cochlear Implants Int.

[7] Bundesausschuss G (2021) Richtlinie. Hilfsmittel-Richtlinie 1–23

[8] Devesse A, Van Wieringen A, Wouters J (2020). AVATAR assesses speech understanding and multitask costs in ecologically relevant listening situations. Ear Hear.

[9] DGHNO-KHC (2021) Weißbuch Cochlea-Implantat (CI)-Versorgung. DGHNO-KHC 2:1–23

[10] Dillier N, Lai WK (2015). Speech intelligibility in various noise conditions with the nucleus® 5 CP810 sound processor. Audiol Res.

[11] Dreschler WA, Verschuure H, Ludvigsen C, Westermann S (2001). ICRA noises: artificial noise signals with speech-like spectral and temporal properties for hearing instrument assessment. Int J Audiol.

[12] Francart T, van Wieringen A, Wouters J (2011). Comparison of fluctuating maskers for speech recognition tests. Int J Audiol.

[13] Goudey B, Plant K, Kiral I, Jimeno-Yepes A, Swan A, Gambhir M, Büchner A, Kludt E, Eikelboom RH, Sucher C, Gifford RH, Rottier R, Anjomshoa H (2021). A multicenter analysis of factors associated with hearing outcome for 2,735 adults with cochlear implants. Trends Hear.

[14] Hahlbrock K (1953). Speech audiometry and new word-tests. Arch Ohren Nasen Kehlkopfheilkd.

[15] Hey M, Böhnke B, Mewes A, Munder P, Mauger SJ, Hocke T (2021). Speech comprehension across multiple CI processor generations: Scene dependent signal processing. Laryngoscope Invest Otolaryngol.

[16] Hey M, Hocke T, Ambrosch P (2018). Sprachaudiometrie und Datalogging bei CI-Patienten: Überlegungen zu geeigneten Sprachpegeln. HNO.

[17] Hey M, Hocke T, Böhnke B, Mauger SJ (2019). ForwardFocus with cochlear implant recipients in spatially separated and fluctuating competing signals–introduction of a reference metric. Int J Audiol.

[18] Hey M, Hocke T, Mauger S, Müller-Deile J (2016). A clinical assessment of cochlear implant recipient performance: implications for individualized map settings in specific environments. Eur Arch Oto-Rhino-Laryngol.

[19] Holden LK, Finley CC, Firszt JB, Holden TA, Brenner C, Potts LG, Gotter BD, Vanderhoof SS, Mispagel K, Heydebrand G, Skinner MW (2013). Factors affecting open-set word recognition in adults with cochlear implants. Ear Hear.

[20] Holube I, Fredelake S, Vlaming M, Kollmeier B (2010). Development and analysis of an international speech test signal (ISTS). Int J Audiol.

[21] Hoppe U, Hey M (2021). Von der Stimmgabel zum 7T MRT – Der Einsatz objektiver Verfahren in der Audiologie. Z Med Phys.

[22] Hoppe U, Hocke T, Hast A, Iro H (2019). Maximum preimplantation monosyllabic score as predictor of cochlear implant outcome. HNO.

[23] Hoth S, Müller-Deile J (2009). Audiologische Rehabilitation von Kochleaimplantat-Trägern. HNO.

[24] ISO 8253‑3 (2012) ISO 8253-3: Acoustics—Audiometric test methods—Part 3 : Speech audiometry. Int Organ Stand 1–31. 10.31030/1861048

[25] Keidser G, Naylor G, Brungart DS, Caduff A, Campos J, Carlile S, Carpenter MG, Grimm G, Hohmann V, Holube I, Launer S, Lunner T, Mehra R, Rapport F, Slaney M, Smeds K (2020). The quest for ecological validity in hearing science: what it is, why it matters, and how to advance it. Ear Hear.

[26] Kropp MH, Hocke T, Agha-Mir-Salim P, Müller A (2021). Evaluation of a synthetic version of the digits-in-noise test and its characteristics in CI recipients. Int J Audiol.

[27] Krueger B, Joseph G, Rost U, Strauss-Schier A, Lenarz T, Buechner A (2008). Performance groups in adult cochlear implant users: speech perception results from 1984 until today. Otol Neurotol.

[28] Lailach S, Neudert M, Zahnert T (2021). Update Cochlea-Implantation: Indikationsstellung und Operation. Laryngorhinootologie.

[29] Laszig R, Lehnhardt E (1987). Cochlear implant. Ein elektronische Hörprothese. Dtsch Ärztebl.

[30] Mauger SJ, Warren CD, Knight MR, Goorevich M, Nel E (2014). Clinical evaluation of the Nucleus 6 cochlear implant system: performance improvements with SmartSound iQ. Int J Audiol.

[31] Meister H (2019). Speech comprehension and cognitive performance in acoustically difficult situations. HNO.

[32] Mrowinski D, Scholz G, Steffens T (2017). Audiometrie.

[33] Müller-Deile J, Kortmann T, Hoppe U, Hessel H, Morsnowski A (2009). Improving speech comprehension using a new cochlear implant speech processor. HNO.

[34] Plesch J, Ernst BP, Strieth S, Rader T (2019). A psychoacoustic application for the adjustment of electrical hearing thresholds in cochlear implant patients. PLoS ONE.

[35] Plontke SK, Girndt M, Meisner C, Probst R, Oerlecke I, Richter M, Steighardt J, Dreier G, Weber A, Baumann I, Plößl S, Löhler J, Laszig R, Werner JA, Rahne T (2016). Multicenter trial for sudden hearing loss therapy – planning and concept. HNO.

[36] Pollack I, Pickett JM (1957). Cocktail party effect. J Acoust Soc Am.

[37] Pyschny V, Landwehr M, Hahn M, Walger M, von Wedel H, Meister H (2011). Bimodal hearing and speech perception with a competing talker. J Speech Lang Hear Res.

[38] Rader T, Doms P, Adel Y, Weissgerber T, Strieth S, Baumann U (2018). A method for determining precise electrical hearing thresholds in cochlear implant users. Int J Audiol.

[39] Rader T, Fastl H, Baumann U (2013). Speech perception with combined electric-acoustic stimulation and bilateral cochlear implants in a multisource noise field. Ear Hear.

[40] Spriet A, Van Deun L, Eftaxiadis K, Laneau J, Moonen M, Van Dijk B, Van Wieringen A, Wouters J (2007). Speech understanding in background noise with the two-microphone adaptive beamformer BEAM^TM^ in the nucleus Freedom^TM^ cochlear implant system. Ear Hear.

[41] Steffens T (2017). Die systematische Auswahl von sprachaudiometrischen Verfahren. HNO.

[42] Volleth N, Hast A, Lehmann EK, Hoppe U (2018). Subjektive Hörverbesserung durch Cochleaimplantatversorgung. HNO.

[43] Wagener KC, Brand T, Wagener C (2009). Sentence intelligibility in noise for listeners with normal hearing and hearing impairment: Influence of measurement procedure and masking parameters La inteligibilidad de frases en silencio para sujetos con audición nor. Int J Audiol.

[44] Weissgerber T, Stöver T, Baumann U (2019). Speech perception in noise: Impact of directional microphones in users of combined electric-acoustic stimulation. PLoS One.

[45] Wolfe J, Parkinson A, Schafer EC, Gilden J, Rehwinkel K, Mansanares J, Coughlan E, Wright J, Torres J, Gannaway S (2012). Benefit of a commercially available cochlear implant processor with dual-microphone beamforming: a multi-center study. Otol Neurotol.

[46] Zöllner F, Hahlbrock KH (1957). Geleitwort. Sprachaudiometrie: Grundlagen und praktische Anwendung einer Sprachaudiometrie für das deutsche Sprachgebiet.

